# Supplementary material for: A genetic toolkit for the human gut bacterium Mediterraneibacter gnavus identifies capsular polysaccharides as a competitive colonization factor
Source: Nat Commun. 2026 Mar 12;17:3855. doi: 10.1038/s41467-026-69022-x (PMC13121745; doi:10.1038/s41467-026-69022-x)
Supplement: Supplementary file 1 — Supplementary Information [file 41467_2026_69022_MOESM1_ESM.pdf]

Supplementary information

A genetic toolkit for the human gut bacterium *Mediterraneibacter gnavus* identifies capsular polysaccharides as a competitive colonization factor

Nozomu Obana[1,2]*, Gaku Nakato[3,4], Nobuhiko Nomura[2,5,6], Shinji Fukuda[1,2,3,4,7]*

1. Transborder Medical Research Center, Institute of Medicine, University of Tsukuba, Tsukuba, Japan.
2. Microbiology Research Center for Sustainability (MiCS), University of Tsukuba, Tsukuba, Japan.
3. Gut Environmental Design Group, Kanagawa Institute of Industrial Science and Technology, Kawasaki, Japan.
4. Innovative Microbiome Therapy Research Center, Juntendo University Graduate School of Medicine, Tokyo, Japan.
5. Institute of Life and Environmental Sciences, University of Tsukuba, Tsukuba, Japan.
6. Tsukuba Institute for Advanced Research, University of Tsukuba, Tsukuba, Japan
7. Institute for Advanced Biosciences, Keio University, Tsuruoka, Japan

***Corresponding Authors:**

Shinji Fukuda, sfukuda@sfc.keio.ac.jp

Nozomu Obana, obana.nozomu.gb@u.tsukuba.ac.jp

Contents:

Supplementary Figures 1 to 10

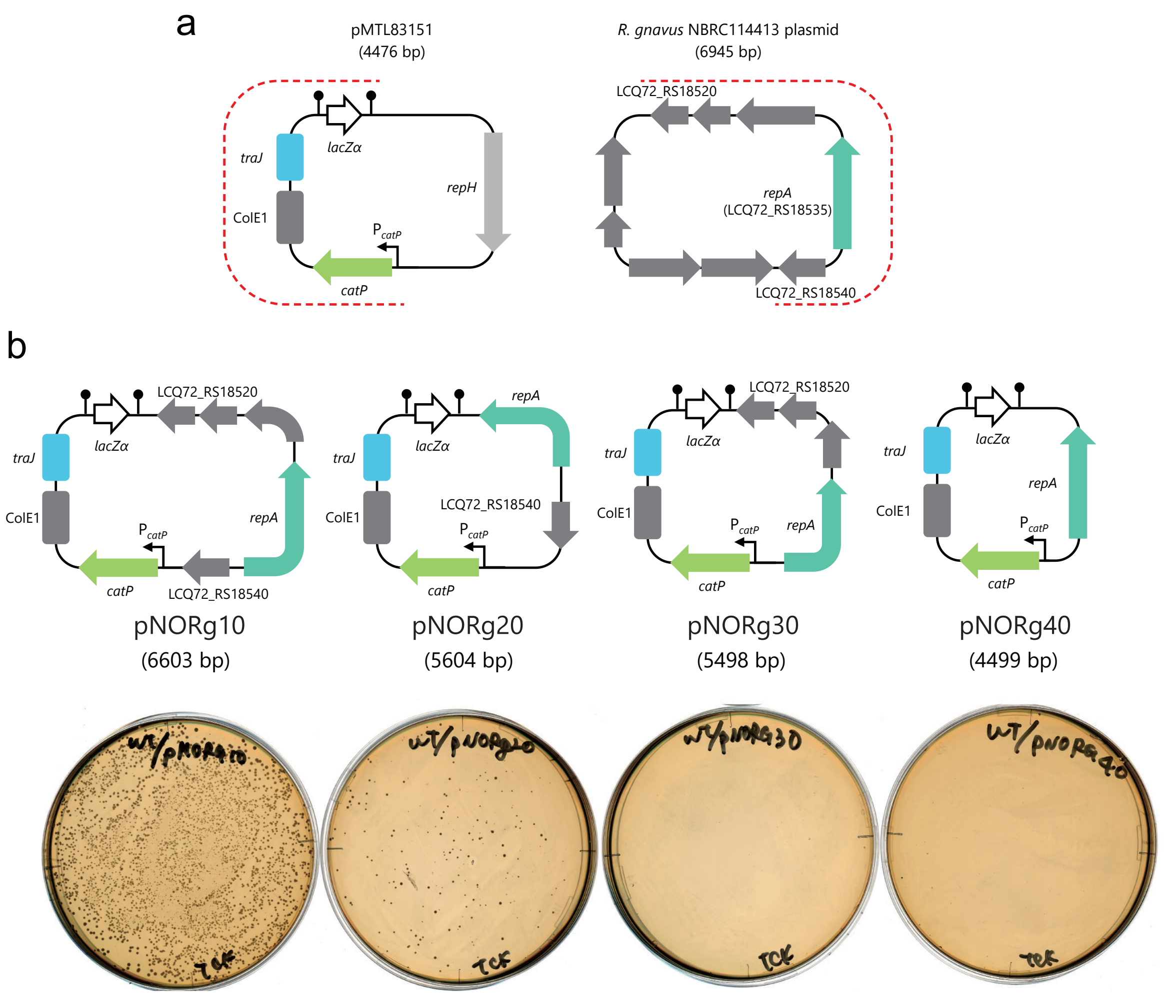

Supplementary Figure 1. Establishment of an *M. gnavus-E. coli* shuttle vector system.

a Schematics of pMTL83151 and an indigenous plasmid in *M. gnavus* NBRC 114413. We used the DNA sequence, indicated by red dot lines, for the construction of the shuttle plasmid, pNORg10. **b** Schematics of pNORg plasmid series. The bottom images show colonies that emerged on selective plates after conjugation. pNORg10 represents the highest conjugation efficiency.

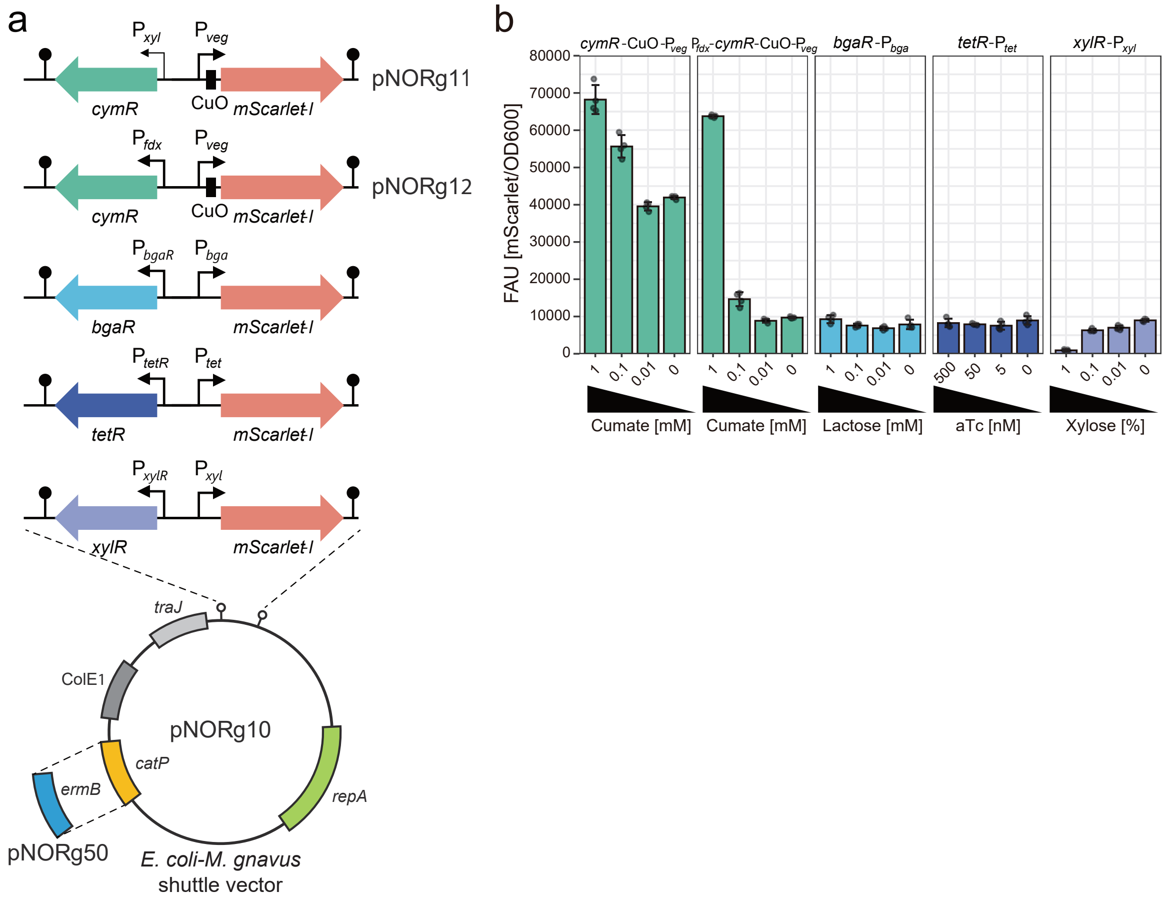

Supplementary Figure 2. Inducible promoter system suitable for *M. gnavus*.

a Several inducible promoters were cloned upstream of the *mScarlet-I* gene in pNORg10. **b** Promoter reporter assay using the shuttle plasmids. Cells were grown to the late exponential phase and exposed to oxygen for at least 90 min to facilitate the maturation of the fluorescent protein. The means ± SD of fluorescent intensities normalized by optical density at 600 nm (OD600), obtained from technical quadruplicates, are shown. Similar results were obtained in three independent experiments.

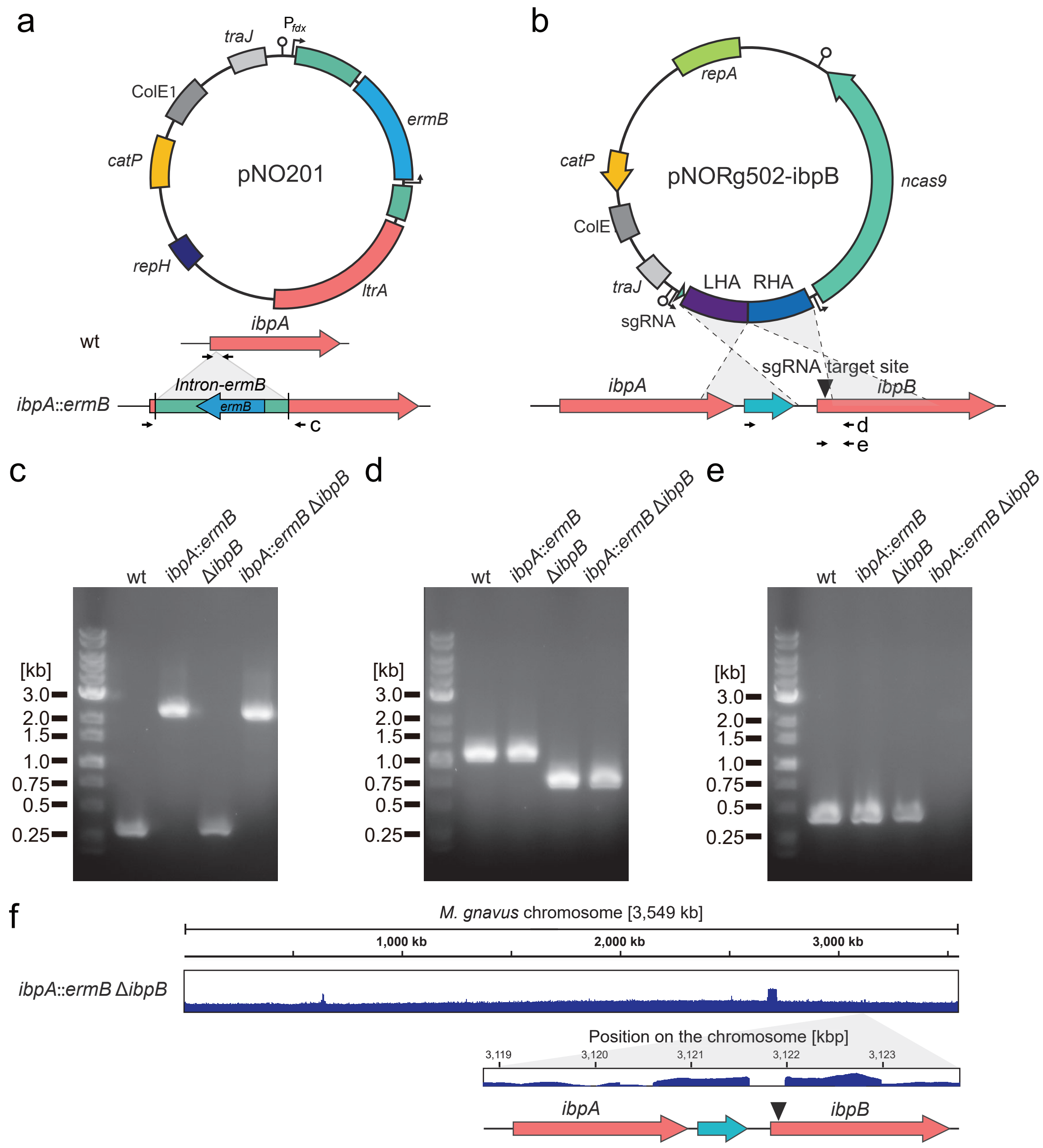

Supplementary Figure 3. Confirmation of *ibpA and ibpB* gene mutants in *M. gnavus*.

a A schematic of the plasmid for the *ibpA* gene disruptant construction. Arrows represent the annealing positions of each primer used for diagnostic PCR. **b** A schematic of the plasmid for constructing an *ibpB* mutant using CRISPR-Cas9. The plasmid includes an sgRNA targeting the chromosomal *ibpB* locus and a repair template spanning the adjacent upstream and downstream regions of *ibpB*. Arrows represent the annealing positions of each primer used for diagnostic PCR. **c** Diagnostic PCR using the primer set indicated as c in Supplementary Fig. 3a. The *ibpA*::*erm* mutant represented larger PCR products than wt. **d** Diagnostic PCR using the primer set indicated as d in Supplementary Fig. 3b. The Δ*ibpB* mutant represented smaller PCR products than wt. **e** Diagnostic PCR using the primer set indicated as e in Supplementary Fig. 3b. The *ibpA*::*erm* Δ*ibpB* double mutant represented no PCR products. In the Δ*ibpB* single mutant, primers annealing to the *ibpA* region, which possesses a sequence similar to *ibpB*, likely amplify the PCR product. **f** The number of mapped reads obtained the genome sequencing of the *ibpA*::*erm* Δ*ibpB* double mutant is plotted on the chromosome of *M. gnavus* ATCC29149. An enlarged image in gene locus including *ibpA* and *ibpB* is shown at the bottom.

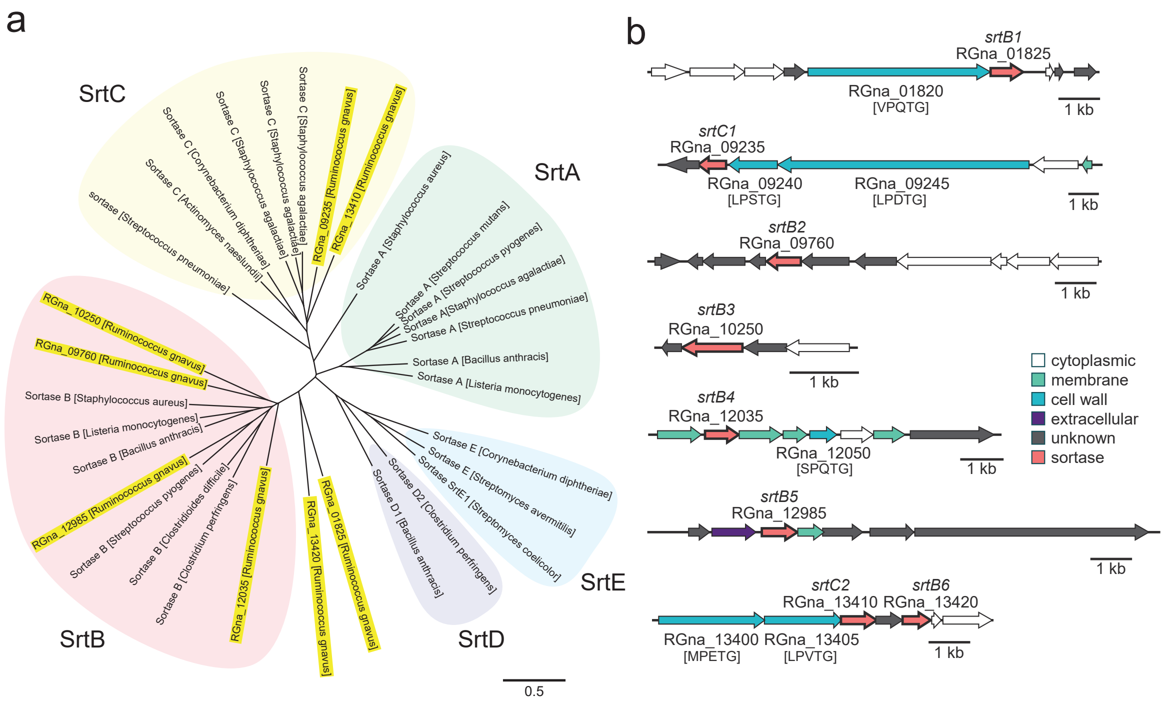

Supplementary Figure 4. Phylogeny and genetic contexts of sortase genes.

a A phylogenetic tree of sortase proteins in *M. gnavus* ATCC 29149 and representative Gram-positive bacteria. Amino acid sequences were aligned by MUSCLE alignment, and the phylogenetic tree was generated using Geneious Tree Builder. **b** Genetic contexts of each sortase gene. Potential sortase target genes located near the sortase genes and possessing putative C-terminal cell wall sorting signals are shown. Colors indicate the subcellular localization of each protein, as predicted by PSORTdb [1].

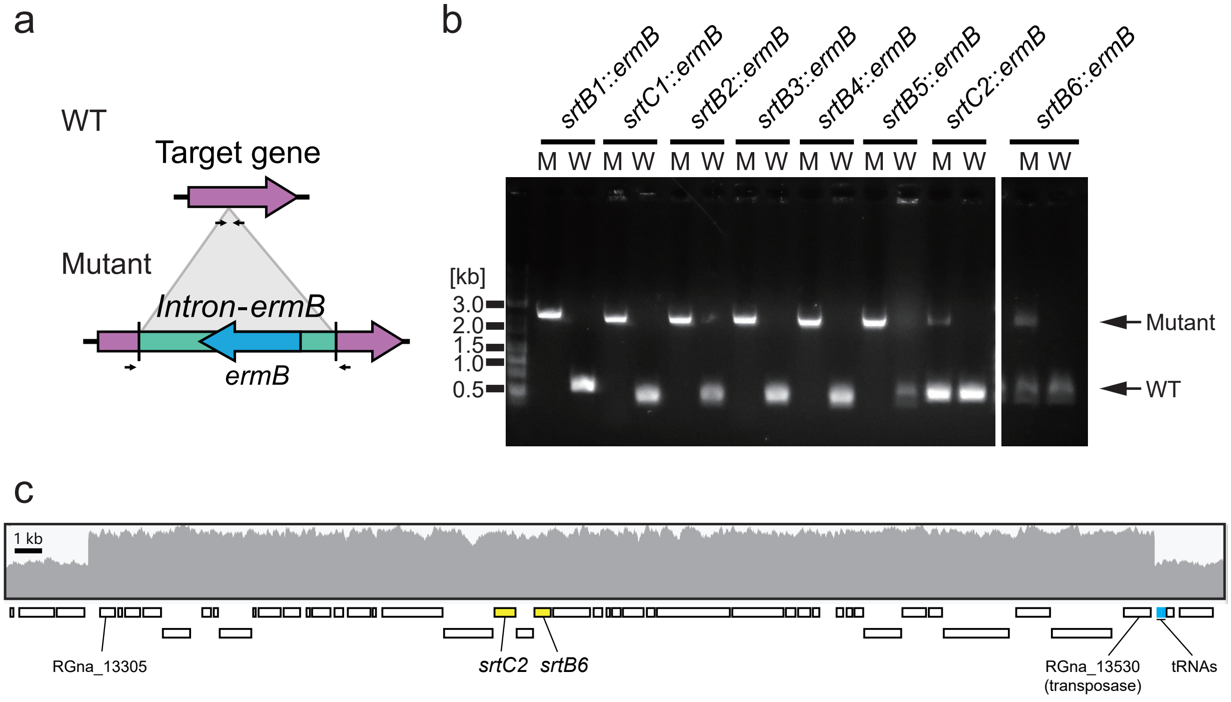

Supplementary Figure 5. Confirmation of sortase gene disruptants in *M. gnavus*.

a Schematics of genes targeted by introns. Small arrows indicate the primers used for colony-directed PCR to confirm the gene disruption. **b** Electrophoresis images of the colony-directed PCR. Upper and lower DNA signals indicate PCR products derived from disruptant mutants and the wild-type, respectively. **c** Screenshot of a genome browser. Genomic DNA was isolated from wild-type *M. gnavus* and used for genome resequencing analysis. The sequence depth in the genome locus containing RGna_13305 to RGna_13530, which includes the *srtC2* and *srtB6* genes, suggests duplication. RGna_13530, a putative transposase gene, is encoded just upstream of tRNA genes, a typical insertion site of mobile genetic elements (MGEs), suggesting that this duplicated region may be an MGE.

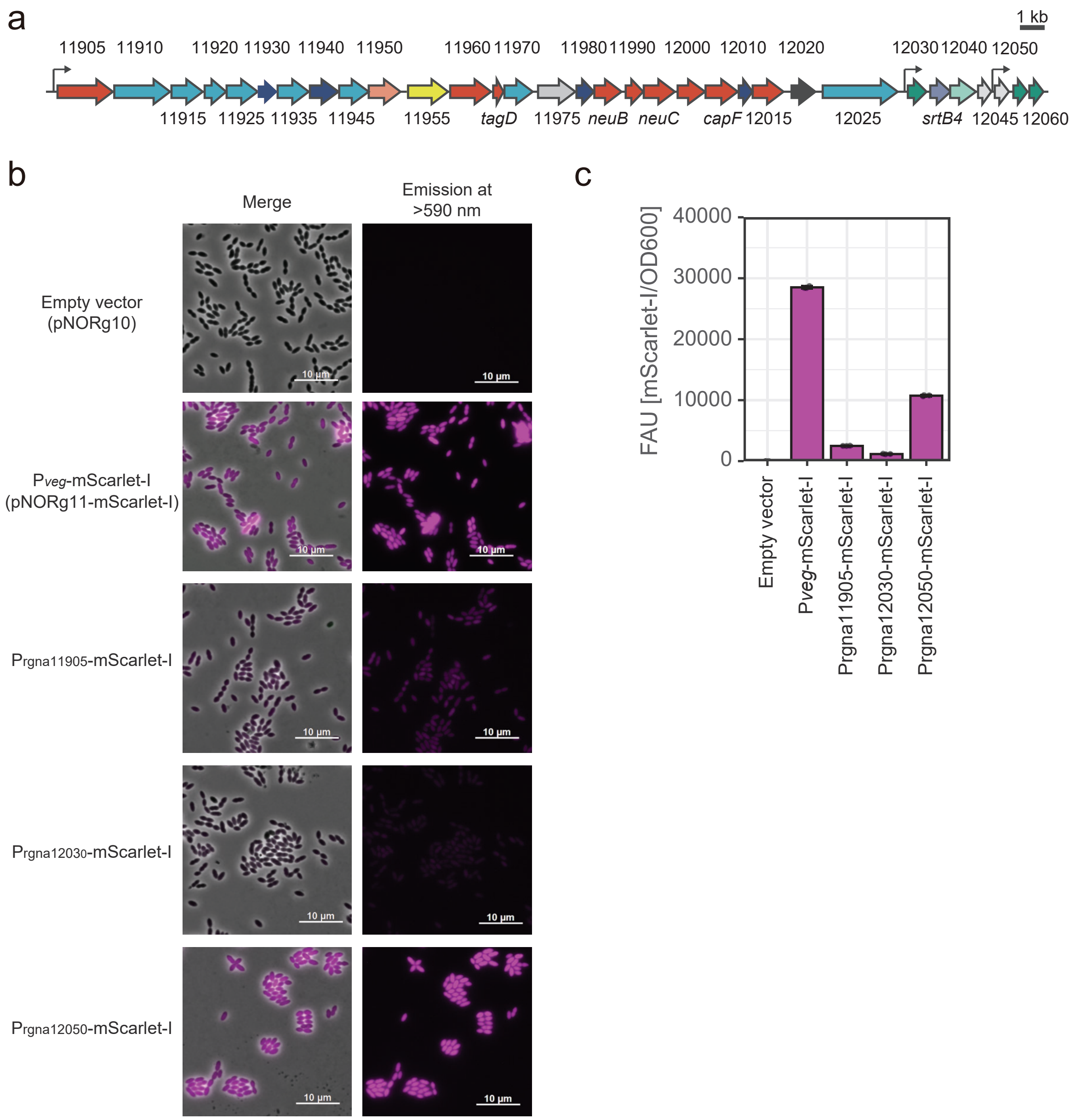

Supplementary Figure 6. Fluorescent reporter analysis of CPS gene promoters in *M. gnavus*.
a Schematics of the predicted promoters in the CPS gene cluster. Three promoters located upstream of RGna_11905, RGna_12030, and RGna_12050 are shown by bent arrows. **b** and **c** Fluorescent reporter analysis of each promoter. Cells were grown for 6 hours to reach the late-exponential phase and then exposed to oxygen for 90 minutes to mature the fluorescent protein, mScarlet-I. Empty vector and pNORg11-mScarlet-I, including the constitutive promoter P_{veg}, were used as negative and positive controls, respectively. Fluorescent microscope images (**b**) and the means ± SD of fluorescent intensities normalized by optical density at 600 nm (OD600), obtained from technical triplicates (**c**), are shown. Similar results were obtained in two independent experiments.

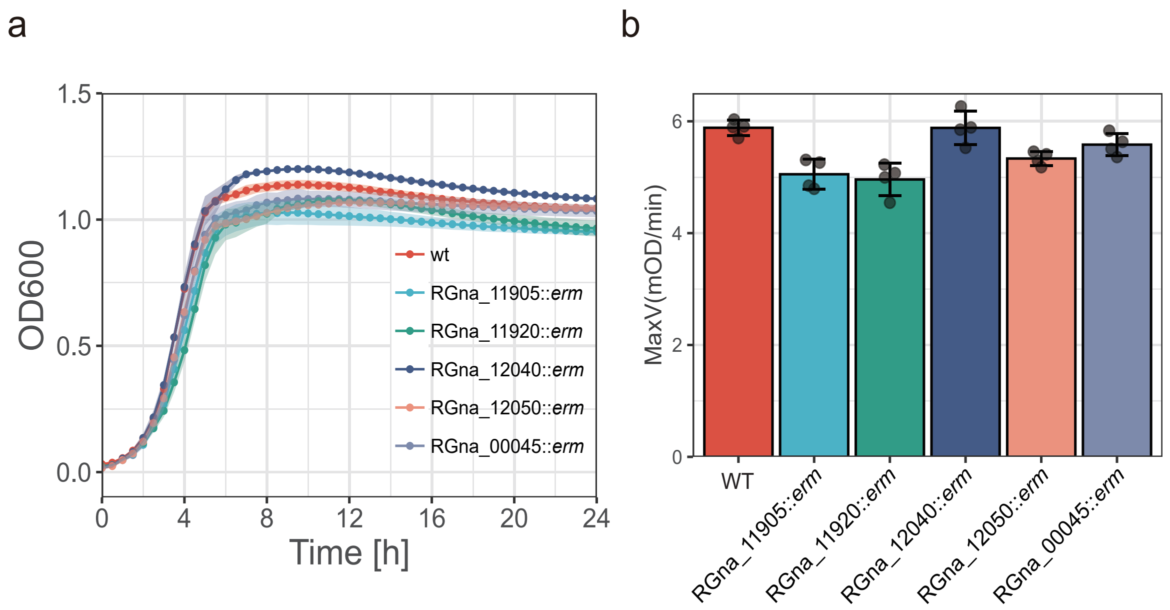

Supplementary Figure 7. Growth curve of CPS gene mutants in *M. gnavus*.

Growth kinetics (a) and maximum velocities (b) of mutant strains of the CPS gene cluster are shown. The means ± SD obtained from 4 replicates are indicated. We confirmed reproducibility through an independent experiment.

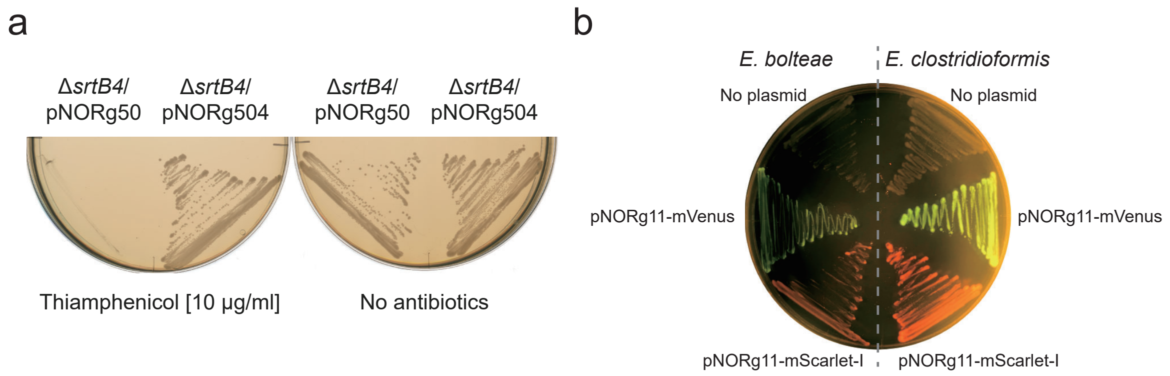

Supplementary Figure 8. Potential applications of the plasmids

a Curing of the CRISPR plasmid. pNORg50 (Erm^R) was introduced into Δ*srtB4* harboring the CRISPR plasmid (pNORg504 (Tm^R)). The loss of thiamphenicol resistance in the resulting strain indicates the curing of the CRISPR plasmid. **b** Fluorescence tagging in Lachnospiraceae strains other than *M. gnavus*. The plasmids pNORg11-mVenus and pNORg11-mScarlet-I were introduced into *E. bolteae* and *E. clostridioformis*. The resulting strains exhibited green/yellow or red fluorescence under blue LED illumination.

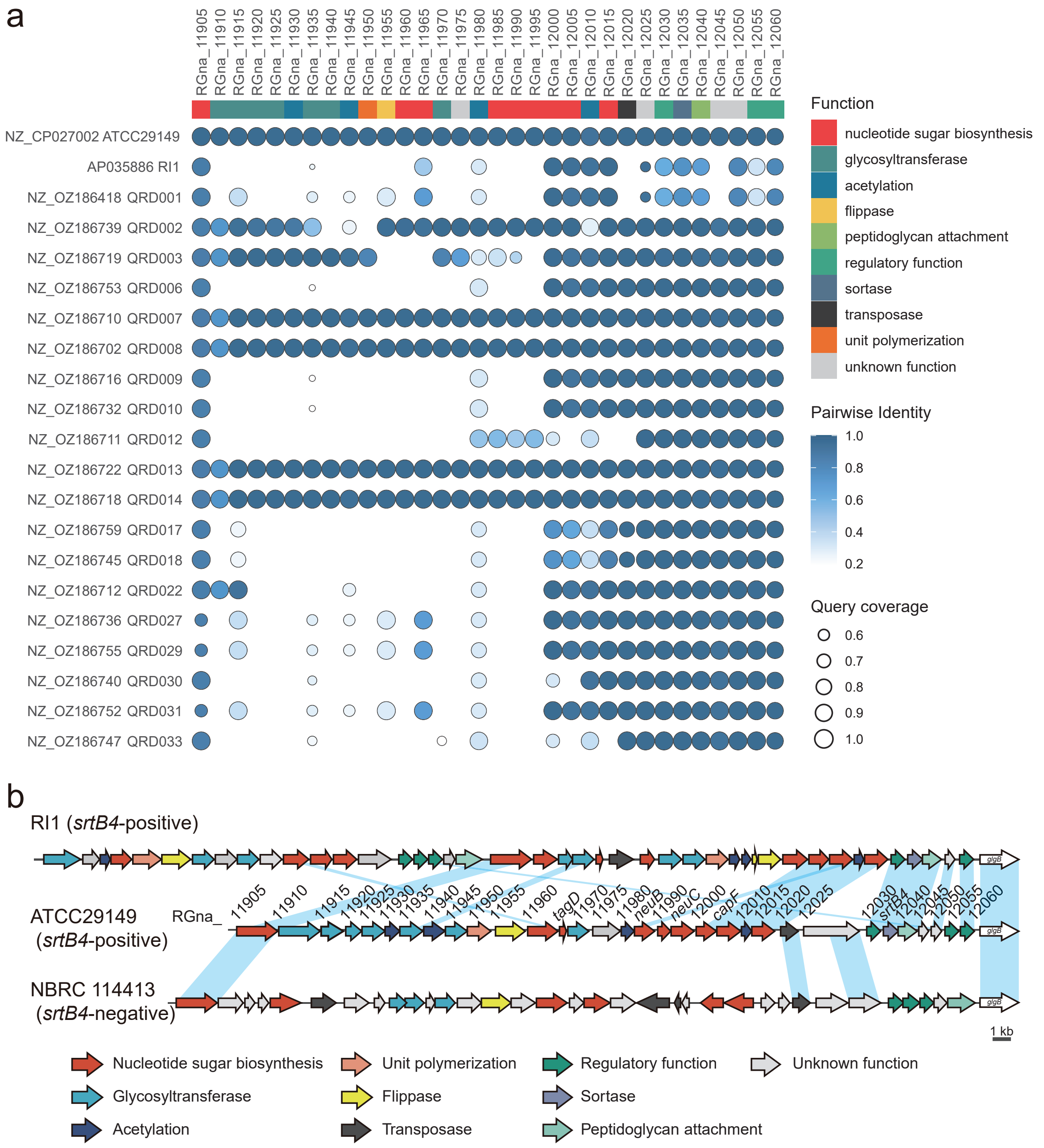

Supplementary Figure 9. Conservation of the entire CPS gene cluster of *M. gnavus* ATCC 29149.

a The conservation and identities of the genes comprising the entire CPS gene cluster of the ATCC 29149 strain were compared with those of *srtB4* homologue-positive isolated strains available in the public database using BLASTn. Pairwise identities and coverage for each gene are shown as a heatmap and circle sizes, respectively. Genes with a similar predicted function are shown in the same color. **b** Genetic contexts of the CPS locus in ATCC 29149, RI1 and NBRC 114413 strains. The gene loci are derived from the upstream of the *glgB* gene, which is highly conserved in all *M. gnavus* strains. Genes with a similar predicted function are shown in the same color. Genes with shared sequences across strains are highlighted in blue.

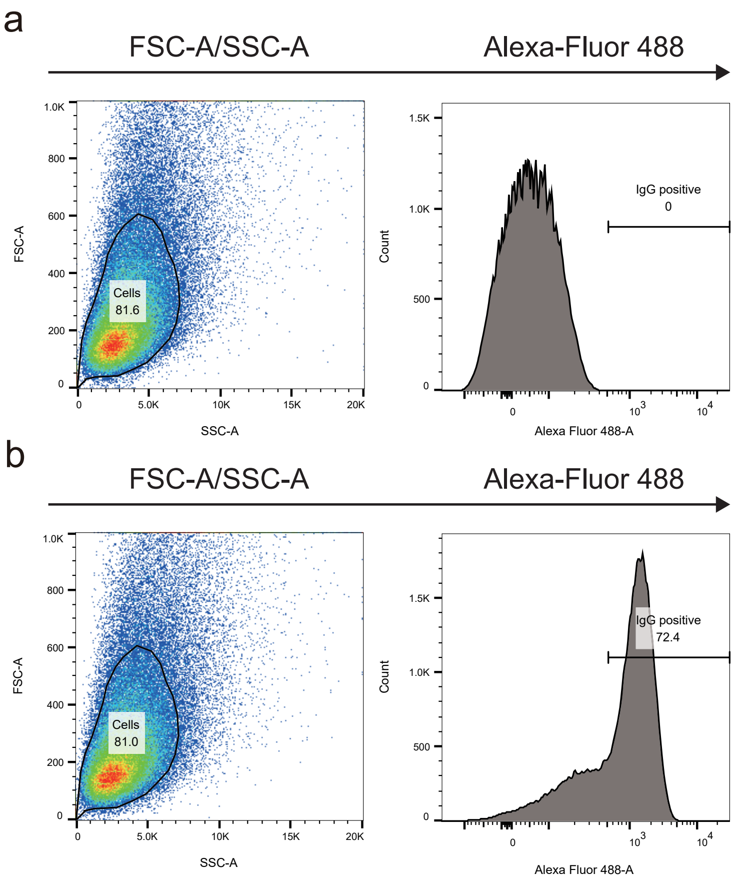

Supplementary Figure 10. Gating strategies in flow cytometry analysis

Representative plots of the gating strategies for wild-type strains without antibody staining (**a**) and with Alexa Fluor 488-conjugated mouse IgG (**b**). Bacterial cell populations were gated by FSC-A/SSC-A. IgG-bound cell populations were quantified by comparison with the unstained control.

Supplementary Reference

1. Peabody MA, Laird MR, Vlasschaert C, Lo R, Brinkman FS. PSORTdb: expanding the bacteria and archaea protein subcellular localization database to better reflect diversity in cell envelope structures. *Nucleic Acids Res* **44**, D663–668 (2016).
