# Supplementary material for: A genetic toolkit for the human gut bacterium Mediterraneibacter gnavus identifies capsular polysaccharides as a competitive colonization factor
Source: Nat Commun. 2026 Mar 12;17:3855. doi: 10.1038/s41467-026-69022-x (PMC13121745; doi:10.1038/s41467-026-69022-x)
Supplement: Supplementary file 2 — Description of Additional Supplementary Files [file 41467_2026_69022_MOESM2_ESM.pdf]

Description of Additional Supplementary Files

Supplementary Data 1. Bacterial strains used in this study.

Supplementary Data 2. Plasmids used in this study.

Supplementary Data 3. Oligonucleotides used in this study.

Supplementary Data 4. The DNA sequence cloned into the shuttle plasmid, including the replicon.
