# Supplementary material for: A genetic toolkit for the human gut bacterium Mediterraneibacter gnavus identifies capsular polysaccharides as a competitive colonization factor
Source: Nat Commun. 2026 Mar 12;17:3855. doi: 10.1038/s41467-026-69022-x (PMC13121745; doi:10.1038/s41467-026-69022-x)
Supplement: Supplementary file 4 — Reporting Summary [file 41467_2026_69022_MOESM4_ESM.pdf]

nature portfolio

Corresponding author(s): Shinji Fukuda

Last updated by author(s): Jan 13, 2026

Reporting Summary

Nature Portfolio wishes to improve the reproducibility of the work that we publish. This form provides structure for consistency and transparency in reporting. For further information on Nature Portfolio policies, see our Editorial Policies and the Editorial Policy Checklist.

Statistics

For all statistical analyses, confirm that the following items are present in the figure legend, table legend, main text, or Methods section.

n/a	Confirmed	
☐	☒	The exact sample size (n) for each experimental group/condition, given as a discrete number and unit of measurement
☐	☒	A statement on whether measurements were taken from distinct samples or whether the same sample was measured repeatedly
☐	☒	The statistical test(s) used AND whether they are one- or two-sided<br>*Only common tests should be described solely by name; describe more complex techniques in the Methods section.*
☐	☒	A description of all covariates tested
☐	☒	A description of any assumptions or corrections, such as tests of normality and adjustment for multiple comparisons
☐	☒	A full description of the statistical parameters including central tendency (e.g. means) or other basic estimates (e.g. regression coefficient) AND variation (e.g. standard deviation) or associated estimates of uncertainty (e.g. confidence intervals)
☐	☒	For null hypothesis testing, the test statistic (e.g. F, t, r) with confidence intervals, effect sizes, degrees of freedom and P value noted<br>*Give P values as exact values whenever suitable.*
☒	☐	For Bayesian analysis, information on the choice of priors and Markov chain Monte Carlo settings
☒	☐	For hierarchical and complex designs, identification of the appropriate level for tests and full reporting of outcomes
☒	☐	Estimates of effect sizes (e.g. Cohen's d, Pearson's r), indicating how they were calculated

Our web collection on statistics for biologists contains articles on many of the points above.

Software and code

Policy information about availability of computer code

Data collection	No custom algorithms or software were not used in this study
Data analysis	Rstudio ver. 2023.06.0 was used for statistical analysis. Geneious Prime (2025.0.3) software was used to analyze genomes.

For manuscripts utilizing custom algorithms or software that are central to the research but not yet described in published literature, software must be made available to editors and reviewers. We strongly encourage code deposition in a community repository (e.g. GitHub). See the Nature Portfolio guidelines for submitting code & software for further information.

Data

Policy information about availability of data

All manuscripts must include a data availability statement. This statement should provide the following information, where applicable:

- Accession codes, unique identifiers, or web links for publicly available datasets
- A description of any restrictions on data availability
- For clinical datasets or third party data, please ensure that the statement adheres to our policy

The manuscript has been included the Data Availability statement including the information requested. The data supporting the findings of this study are available within this article and the Supporting Information. The source data are provided with this paper as a Source data file. Plasmids are available from Addgene (Plasmid ID, 249340-249345). Whole genome sequence data generated in this study have been deposited in the DDBJ Sequence Read Archive (DRA) under accession number XXXXX.

Research involving human participants, their data, or biological material

Policy information about studies with human participants or human data. See also policy information about sex, gender (identity/presentation), and sexual orientation and race, ethnicity and racism.

Reporting on sex and gender	n/a
Reporting on race, ethnicity, or other socially relevant groupings	n/a
Population characteristics	n/a
Recruitment	n/a
Ethics oversight	n/a

Note that full information on the approval of the study protocol must also be provided in the manuscript.

Field-specific reporting

Please select the one below that is the best fit for your research. If you are not sure, read the appropriate sections before making your selection.

☒ Life sciences ☐ Behavioural & social sciences ☐ Ecological, evolutionary & environmental sciences

For a reference copy of the document with all sections, see nature.com/documents/nr-reporting-summary-flat.pdf

Life sciences study design

All studies must disclose on these points even when the disclosure is negative.

Sample size	No statistical method was used to determine the sample size. Sample sizes in this study is described in materials and methods section or Figure legends.
Data exclusions	No data were excluded.
Replication	We obtained the reproducibility of each experiment from at least 2 independent experiments.
Randomization	Samples were not randomized.
Blinding	Investigators were not blinded in this study.

Reporting for specific materials, systems and methods

We require information from authors about some types of materials, experimental systems and methods used in many studies. Here, indicate whether each material, system or method listed is relevant to your study. If you are not sure if a list item applies to your research, read the appropriate section before selecting a response.

Materials & experimental systems

n/a	Involved in the study	
☐	☒	Antibodies
☐	☒	Eukaryotic cell lines
☒	☐	Palaeontology and archaeology
☐	☒	Animals and other organisms
☒	☐	Clinical data
☒	☐	Dual use research of concern
☒	☐	Plants

Methods

n/a	Involved in the study	
☒	☐	ChIP-seq
☐	☒	Flow cytometry
☒	☐	MRI-based neuroimaging

Antibodies

Antibodies used	We used mouse IgG Alexa Fluor 488 conjugates (Thermo Fisher Scientific, A-10631; lot number, 2400911).
Validation	Validation is not relevant to this work. We confirmed that the IgG did not bind to R. gnavus ibpA and ibpB double mutant strain.

Eukaryotic cell lines

Policy information about cell lines and Sex and Gender in Research

Cell line source(s)	We purchased J774.1 mouse macrophage like cells from RIKEN BRC.
Authentication	The cell line used in this study was not authenticated.
Mycoplasma contamination	The cell line has been confirmed to be free of mycoplasma contamination.
Commonly misidentified lines (See ICLAC register)	n/a

Animals and other research organisms

Policy information about studies involving animals; ARRIVE guidelines recommended for reporting animal research, and Sex and Gender in Research

Laboratory animals	BALB/c Cr mice
Wild animals	n/a
Reporting on sex	We used male mice for the DSS treatment experiment and female mice for the competitive colonization assay.
Field-collected samples	n/a
Ethics oversight	The animal experiment committee in University of Tsukuba approved mouse experiments in this study.

Note that full information on the approval of the study protocol must also be provided in the manuscript.

Plants

Seed stocks	n/a
Novel plant genotypes	n/a
Authentication	n/a

Flow Cytometry

Plots

Confirm that:

- [x] The axis labels state the marker and fluorochrome used (e.g. CD4-FITC).
- [x] The axis scales are clearly visible. Include numbers along axes only for bottom left plot of group (a 'group' is an analysis of identical markers).
- [x] All plots are contour plots with outliers or pseudocolor plots.
- [x] A numerical value for number of cells or percentage (with statistics) is provided.

Methodology

Sample preparation	Sample preparation is described in materials and methods section.
Instrument	FACS Aria II flow cytometer (BD Biosciences).
Software	Data were analyzed using FlowJo V10.8.
Cell population abundance	We analyzed bacterial cells collected from liquid cultures and all cells to quantified the population of IgG-binding cells.
Gating strategy	FSC/SSC gate was used to remove debris, and dominant population was selected, which contains >90% of total events.

- [x] Tick this box to confirm that a figure exemplifying the gating strategy is provided in the Supplementary Information.
